# Corn Seed Freezing Damage Identification of Different Sides Based on Hyperspectral Imaging and SPA-2DCOS Fusion Algorithm

**DOI:** 10.3390/molecules30102178

**Published:** 2025-05-15

**Authors:** Jun Zhang, Limin Dai, Ruiyuan Zhuang

**Affiliations:** 1 School of Mechanical and Electrical Engineering, Jiaxing Nanhu University, 572 Yuexiu South Road, Jiaxing 314001, China; 11613006@zju.edu.cn; 2 School of Agricultural Engineering, Jiangsu University, 301 Xuefu Road, Zhenjiang 212013, China; lmdai@ujs.edu.cn

**Keywords:** freeze-damaged corn seed, endosperm, embryo, hyperspectral imaging, SPA, 2DCOS, feature wavelength fusion

## Abstract

In order to improve the utilization efficiency of corn seeds and meet the demand of single-seed seeding technology in agriculture, this study was conducted to explore the effect of freezing damage detection on the endosperm and embryo sides of single corn seeds, based on hyperspectral imaging combined with a feature fusion algorithm and a machine learning method. First, hyperspectral image data of the endosperm and embryo sides of three freezing damage categories of corn seeds were collected, and the average spectra of the endosperm part and embryo part were obtained by threshold segmentation. Then, the spectral data were preprocessed (none, SNV, and 5-3 smoothing), and the feature wavelengths were extracted using the feature wavelength extraction algorithm (SPA and 2DCOS). The modeling accuracy results based on the hyperspectral data of the endosperm and embryo sides at the full waveband and feature wavelength (including feature wavelength fusion) were compared and analyzed. In the endosperm side’s freezing damage identification, the SNV+SVM model obtained the highest accuracies of 92.9% and 90.0% with the training set and testing set, based on the full-waveband data. The SNV+SPA-2DCOS+SVM model, based on the feature wavelengths, obtained the highest accuracies of 92.9% and 91.2% with the training set and testing set, respectively. In terms of the embryo side’s freezing damage identification, the results on the embryo side were better than those on the endosperm side. The 5-3 smoothing+LDA model, based on the full-waveband data, achieved the highest accuracy results of 97.7% and 95.9% with the training and testing sets. In the meantime, the none+SPA-2DCOS+LDA model, based on the feature wavelengths, achieved the same highest accuracy results with the training and testing sets. When the fusion algorithm consisting of SPA and 2D-COS was used, the model’s performance on the endosperm side was better than that of the full-waveband analysis with only 19 feature wavelengths, while the recognition effect on embryo side could be achieved with only 15 feature wavelengths. These results provide a theoretical basis for constructing a multi-spectral detection system for the rapid and nondestructive identification of frozen corn seeds.

## 1. Introduction

Maize (*Zea mays* L.) is one of the world’s three main food crops, along with rice and wheat, and it is also a major feed crop. According to the Food and Agriculture Organization of the United Nations (FAO)’s statistics, the global yield of corn was more than 1.16 billion tons in 2022 (https://www.fao.org/faostat/zh/#data/QC, accessed on 17 March 2025). China has the world’s second largest corn-planting area and output, and corn occupies an important position in China’s agricultural production [1].

The Hexi Corridor is an important corn seed production base in China. Because of its geographical location and seasonal climate, this area is often affected by cold waves and freezing damage from September to May each year, with a significant impact on corn seed production. The freezing damage of corn seed has become an agricultural disaster. The quality of seeds exposed to low temperatures will decrease, and the chemical composition inside the seeds will also change, which has a significant impact on seed germination, root growth, and post-emergence growth. The quality of seeds is of vital importance to a series of subsequent stages, such as growth, development, and harvest. Regarding research on factors affecting the freezing damage of corn seeds, scholars have discussed the relationships between seed maturity, water content, freezing temperature, and freezing damage duration and the seed germination rate under different freezing damage conditions [2,3,4,5]. Woltz et al. (2006) studied the effect of water content on the seed germination rate and concluded that when the water content of seeds exceeded about 40%, freezing temperatures of −6 °C and −10 °C had significant effects on the seed germination rate [4]. Zhang et al. (2022) investigated the physicochemical properties and microstructure of corn seeds affected by freezing damage, and their results showed that the deeper the degree of the freezing damage, the lower the germination rate of corn seeds. The changes in seed texture and cell structure reflected the effects of the freezing injury on maize seeds [6].

In actual agricultural production, it is necessary to test freeze-damaged corn seeds, especially through the nondestructive testing of the seed quality. The International Seed Testing Association (ISTA) recommended methods such as the electrical conductivity test for seed viability testing in 1995, but these methods had limitations such as using destructive testing and being time-consuming [7]. In addition, some chemical detection methods require the destruction of seeds, which limits their application. Therefore, there is a lack of rapid and nondestructive methods for the detection of freeze-damaged seeds. The quick and accurate identification of seeds affected by freezing damage has become a major challenge for the fine control of seed quality.

With the development of spectroscopy and its imaging technologies, some scholars have tried to detect frozen corn seeds. Agelet et al. (2012) and Jia et al. (2016) used near-infrared spectroscopy (NIRS) and chemometrics to study the feasibility of analyzing frost-damaged corn seed kernels [8,9]. In Jia et al.’s study, frost-damaged seeds stored at −19.2 °C with a moisture content of 30% were identified using a biomimetic pattern recognition method, and the highest average accuracy of 97% was obtained [9]. Zhang et al. (2022) applied NIRS combined with different preprocessing methods, feature extraction methods, and modeling methods to identify the frozen corn seeds, and the results showed that the KNN model has 99.4% and 100% classification accuracies for the training set and testing set. Although this technology can achieve excellent results, it is hard to determine the characteristics of individual corn seeds [6].

In agricultural production practice, there are some differences in the states of corn seeds on the same ear harvested at the same time when freezing damage occurs. In order to improve the utilization efficiency of seeds and meet the demand of single-seed seeding technology in agriculture, it is important to find a nondestructive testing technology to realize the detection of single corn seeds. As an advanced approach integrating spectral and image technology, hyperspectral imaging (HSI) has a certain research basis in the fields of agricultural products and food testing [10,11,12,13,14,15,16], especially in the nondestructive testing of corn seed quality [17,18,19,20]. This technology can not only accurately locate the specific position of the sample but also extract the spectral information of each part of the sample and use the spectral information to further analyze the regional characteristics. Thus, taking into consideration the differences in the endosperm and the embryo side of corn seeds, this study used HSI technology to collect the hyperspectral image data of corn seeds on the endosperm and embryo sides and compared the identification performance of single seeds.

According to the review of the existing literature, the region of interest (ROI) and its spectral data are usually extracted by an image processing method firstly when processing the hyperspectral image data. Then, steps such as preprocessing [21,22], feature wavelength extraction [23,24,25], and the use of a modeling algorithm [26] are generally applied. The use of different data analysis methods has a significant impact on the modeling results. In the process of algorithm exploration, some researchers use a feature fusion algorithm to model and achieve good results [27,28]. In view of the advantages of two-dimensional correlation analysis (2DCOS) in capturing weak peaks and covering peaks in spectra [29,30], this study will compare the modeling accuracy results of each model after the wavelength fusion process. Given the gaps in existing research, it is interesting to explore SPA-2DCOS wavelength fusion and compare the effectiveness of endosperm and embryo analyses of single seeds.

To summarize, in view of the destructive characteristics of current chemical detection methods, the difficulty of NIRS nondestructive testing technology in detecting individual seeds alone, and the differences between the embryo and endosperm sides of freeze-damaged seeds, this study proposes a method of detecting different sides of these seeds using hyperspectral imaging technology. Furthermore, in order to facilitate the future application of this method in the actual field, this study also discusses the selection and fusion methods of these feature wavelengths, assessing their feasibility for practical applications. Thus, the main objectives of this study were to (1) obtain the single corn seed endosperm and embryo hyperspectral images; (2) establish classification models with different preprocessing methods, wavelength selection methods, and modeling methods; (3) compare the model accuracy results based on full-waveband and feature wavelengths; (4) explore the modeling improvement by wavelength fusion methods and evaluate the optimal model performance.

## 2. Results and Discussion

### 2.1. Original and Preprocessing Spectra

**Figure 1:**
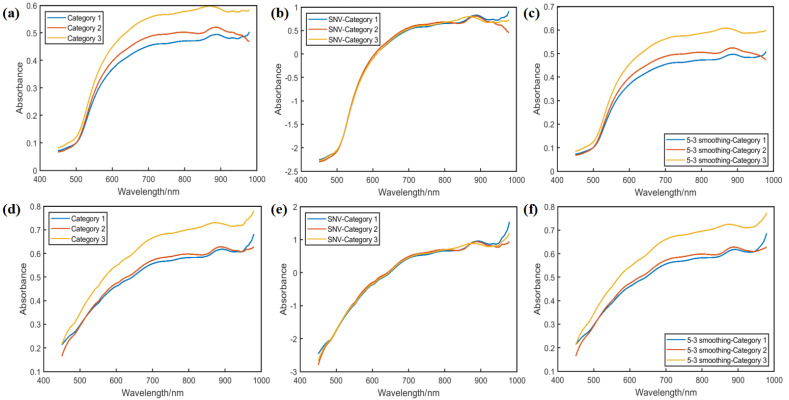
The average spectra of the different freeze-damaged corn seed samples under different preprocessing methods. (**a**–**c**) Original average spectra, SNV preprocessed spectra, and 5-3 smoothing preprocessed spectra in the endosperm. (**d**–**f**) Original average spectra, SNV preprocessed spectra, and 5-3 smoothing preprocessed spectra in the embryo.

Figure 1 shows the original average spectra and preprocessing spectra of the corn seed samples of three freeze-damaged categories in the endosperm (a–c) and the embryo (d–f). Although the general trends of the spectral curves were similar for different categories across the whole waveband range, differences in the reflectance spectral value at a specific wavelength range could also be observed. It can be seen that the reflectance spectral value may be related to the frozen environment, as the unfrozen samples had the lowest spectral value, while the highest spectral value appeared in category 3. Some studies have shown that when seeds are damaged by frost damage, the surface color may be darker; thus, there were spectral differences in the data collected under the irradiation of a light source. In addition, the spectral value of the endosperm-side corn samples was lower than that of the embryo-side samples. This may be because the image on the embryo side is brighter than that of the endosperm side. The existence of these similarities and differences suggested that it was possible to classify different freeze-damaged corn seeds.

### 2.2. Selection of Feature Wavelength

#### 2.2.1. SPA

**Figure 2:**
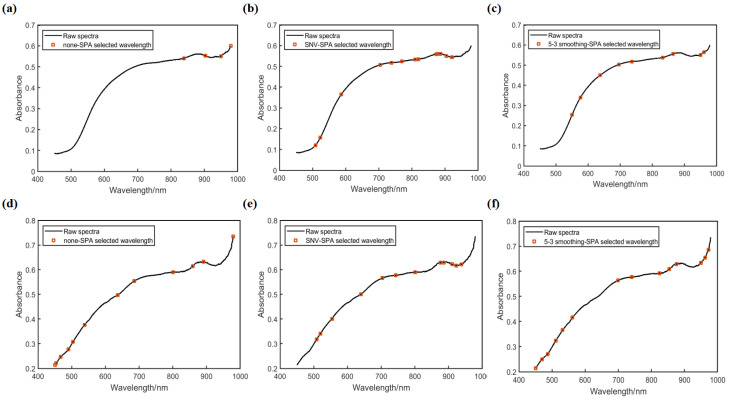
The feature wavelengths obtained by SPA. (**a**–**c**) Original average spectra, SNV preprocessed spectra, and 5-3 smoothing preprocessed spectra in the endosperm. (**d**–**f**) Original average spectra, SNV preprocessed spectra, and 5-3 smoothing preprocessed spectra in the embryo.

Figure 2 shows the feature wavelengths on the endosperm (a–c) and embryo (d–f) side extracted by SPA. It can be seen from the figure that the wavelengths from different preprocessing spectra were located in different positions. The feature wavelengths of each preprocessing algorithm are shown in Table 1. Under SPA, the original spectrum, the SNV preprocessed spectrum, and the 5-3 smoothing preprocessed spectrum of the endosperm obtained 4, 13, and 9 feature wavelengths, respectively, and those of the embryo obtained 12, 12, and 14 feature wavelengths, respectively. From Table 2, the same feature wavelength position appeared at 979 nm with no preprocessing on the endosperm and embryo side, while the same wavelengths in 509 nm and 886 nm were obtained with SNV pretreatment, and that in 949 nm was obtained with 5-3 smoothing pretreatment.

##### 2.2.2. 2DCOS

**Figure 3:**
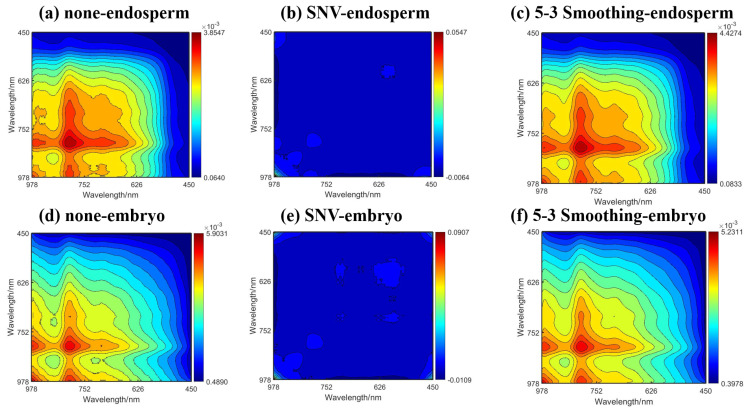
The 2D correlation synchronous spectra. (**a**–**c**) Original average spectrum, SNV preprocessed spectrum, and 5-3 smoothing preprocessed spectrum in the endosperm. (**d**–**f**) Original average spectrum, SNV preprocessed spectrum, and 5-3 smoothing preprocessed spectrum in the embryo.

Figure 3 shows the two-dimensional correlation synchronization spectra on the endosperm (a–c) and embryo (d–f) side of corn seeds. It was found that the original spectrum and the 5-3 smoothing pretreatment spectrum had similar two-dimensional correlation synchronization spectrum results, and the response range of both spectra was mainly concentrated in the range of 700–979 nm, while the response range of the SNV pretreatment spectrum was concentrated in the whole waveband range.

**Figure 4:**
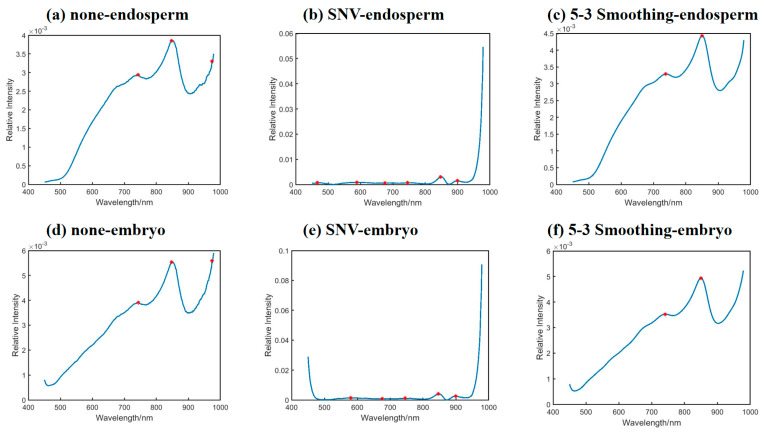
The autocorrelation spectra of 2D correlation synchronous spectra. (**a**–**c**) Original average spectrum, SNV preprocessed spectrum, and 5-3 smoothing preprocessed spectrum in the endosperm. (**d**–**f**) Original average spectrum, SNV preprocessed spectrum, and 5-3 smoothing preprocessed spectrum in the embryo.

Figure 4 shows the autocorrelation peak spectra on the diagonal matrix of the two-dimensional correlation synchronization spectra on the endosperm part (a–c) and the embryo part (d–f) of corn seed samples. Under the 2DCOS method, the original spectrum, SNV preprocessed spectrum, and 5-3 smoothing preprocessed spectrum of the endosperm obtained 3, 6, and 2 feature wavelengths, respectively, and those of the embryo obtained 3, 5, and 2 feature wavelengths, respectively. The feature wavelengths of each preprocessing algorithm are shown in Table 1. From Table 1, the number of wavelengths obtained on the endosperm and embryo side of each preprocessing method were similar, and the position of each wavelength was similar. The same wavelengths in 848 nm and 974 nm were obtained with no pretreatment, those in 676 nm, 745 nm, and 899 nm were obtained with SNV pretreatment, and that in 850 nm was obtained with 5-3 smoothing pretreatment.

Due to the ability of 2DCOS to detect low-response wavelengths [29], it is possible to reveal key feature wavelengths in the field of seed frost damage recognition and classification. Therefore, in this study, wavelengths identified by other feature wavelength extraction algorithms were integrated with those extracted by the 2DCOS method. Finally, the feature wavelengths of the endosperm and embryo obtained by the fusion method are shown in Table 1.

### 2.3. Classification Results on Endosperm Side

Table 2 shows the endosperm-side modeling accuracy based on different preprocessing methods. Firstly, by comparing the performance of the three models, it was found that the accuracy of the LDA and SVM models were higher than the KNN model under various preprocessing conditions. Then, the results of full-band modeling were analyzed, and the results showed that the SVM model based on the full band showed the best performance no matter which preprocessing method was used. Specifically, under the condition of none and 5-3 smoothing preprocessing methods, the SVM model had the highest accuracy of 93.0% and 88.6% of the training set and testing set, respectively. Under the SNV preprocessing method, the highest accuracy of the training set and testing set was increased to 92.9% and 90.0%, respectively, which was better than the results of other preprocessing methods. Further, the modeling results of the feature wavelength extraction algorithm were compared, and the results showed that the model based on SPA was generally superior to the model based on the 2DCOS algorithm. In the case of the SNV+SVM model, the highest accuracy of the training set and testing set reached 92.6% and 90.9%, respectively, which was the best result among all algorithms. Finally, this study evaluated the modeling results of the fusion algorithm SPA-2DCOS and found that the performance of the model after fusion was improved compared with that before the fusion process. In the case of SNV preprocessing combined with the SVM model, the highest accuracy of the training set and testing set reached 92.9% and 91.2%, respectively, which was the best performance in the fusion algorithm.

**Table 2:** The accuracy results of each model under different preprocessing methods on the endosperm side.

Preprocessing	Extraction Methods	KNN		LDA		SVM	
		Training	Testing	Training	Testing	Training	Testing
None	Full-band	85.8%	84.7%	93.4%	88.5%	93.0%	88.6%
	SPA	91.9%	88.5%	92.3%	88.8%	92.6%	88.3%
	2DCOS	91.5%	88.6%	92.2%	88.6%	92.4%	88.6%
	SPA+2DCOS	91.6%	88.5%	92.9%	89.7%	92.9%	88.6%
SNV	Full-band	90.3%	87.5%	93.8%	88.8%	92.9%	90.0%
	SPA	90.2%	86.3%	92.5%	88.5%	92.6%	90.9%
	2DCOS	87.3%	85.8%	89.0%	86.7%	90.2%	87.2%
	SPA+2DCOS	91.0%	87.1%	92.6%	88.6%	92.9%	91.2%
5-3 smoothing	Full-band	85.3%	85.6%	93.7%	88.1%	93.0%	88.6%
	SPA	85.5%	85.0%	93.0%	88.9%	93.0%	88.6%
	2DCOS	77.1%	74.9%	82.5%	81.6%	83.9%	82.2%
	SPA+2DCOS	87.1%	85.8%	93.0%	88.9%	93.0%	88.6%

### 2.4. Classification Results on Embryo Side

Table 3 shows the embryo-side modeling accuracy based on different preprocessing methods. The analysis process was similar to that of the endosperm side. Firstly, the performance of the three models was compared: no matter which preprocessing method was used, the modeling performance of the LDA and SVM models was better than that of the KNN model. Then, the full-band modeling results were analyzed: under the condition of none and SNV preprocessing methods, the SVM model performed best, with the highest accuracy of the training set being 97.3% and 98.1%, and the highest accuracy of the testing set being 94.1% and 95.4%, respectively. Under the 5-3 smoothing preprocessing method, the LDA model had the best performance, and the highest accuracy of the training set and testing set was 97.7% and 95.9%, respectively, which was better than the results obtained under the other two preprocessing methods. Further, the modeling results of the feature wavelength extraction algorithm were compared: the results were consistent with the endosperm part; that is, the modeling performance of SPA was generally better than that of the 2DCOS algorithm, and the performance of the LDA model under none and 5-3 smoothing pretreatment was better than that of the SVM model. Under the condition of SNV pretreatment, the LDA model achieved the best performance, and the highest accuracy results of the training set and testing set were 97.7% and 94.8%. Finally, the modeling results of the SPA-2DCOS fusion algorithm were analyzed, and it was found that the performance of the model after fusion was improved compared with that before fusion. The LDA models without pretreatment and the SNV preprocessing method performed better, and the highest accuracy of the training set and testing set of the LDA models without pretreatment reached 97.5% and 94.9%, respectively, which were the best results.

### 2.5. Model Performance in Optimal Wavelength Selection on Each Side

In the fusion algorithm, the highest accuracy of the training set and testing set reached 92.9% and 91.2%, respectively, for the endosperm side in the SNV pretreatment and the SVM model, while it was 97.5% and 94.9%, respectively, for the embryo side in the none pretreatment and the LDA model. In order to compare the model performance on each side, Table 4 shows the confusion matrix and the sensitivity value of the optimal wavelength selection model on each side. From Table 4, it can be seen that whether it is the result of the endosperm side or the embryo side, category 1 (normal) corn seeds can be detected well. A small part of them are wrongly classified into other categories in the testing set. Seeds in categories 2 (slightly) and 3 (severely) are prone to misclassification. The possible reason for the misclassification of seeds in these two categories is that seeds in both categories have suffered from frost damage. By examining the results of sensitivity, it is still the case that the value of category 1 is higher, the value of category 3 is the lowest, and the value of category 2 is second.

To evaluate the problems in model quality performance that may be caused by the differences in data amounts from the three category datasets, the Accuracy RND of each training and testing set was calculated. For the analysis of the three categories, when their data amounts are all equal, this represents the ideal balanced case for which Accuracy RND is 33.3%. For the endosperm surface, the calculated Accuracy RND of the training set and testing set was 34.9%, while for the embryo surface, the Accuracy RND value was 34.8%, which is very close to 33.3%. As for the ‘Accuracy-Accuracy RND’ value, the ideal balanced case is the one in which the ‘Accuracy-Accuracy RND’ value is 67.7%. After calculation, for the endosperm side, the values of the training set and testing set were 58.0% and 56.3%, respectively, while for the embryo side, the values were 62.7% and 60.1%. It can be seen that these values are close to 66.7%, especially in the case of the embryo side. Therefore, we are not “burdened” with the problem of class imbalance in this study, which also indicates that the quality of the model is relatively high.

Further comparison of the modeling results of the endosperm side and embryo side showed that the classification effect of the embryo side was better. This is because the structure and physiological characteristics of corn seed embryos make them more sensitive to freezing damage [6,31], as they contain high concentrations of metabolically active substances, organelles, and membrane systems, which are vulnerable to damage during freezing, resulting in the loss of membrane function, changes in the seed cortex, and the degeneration and aggregation of biological molecules such as proteins and nucleic acids, affecting the metabolic activity and growth of embryos [6]. In contrast, the endosperm is mainly composed of relatively stable substances such as starch and is less sensitive to freezing damage [31]. From the spectral data, the spectrum of seed embryos is complex, including the characteristic absorption peaks of a variety of biomolecules. Changes in the molecular structure during freezing will cause changes in spectral characteristics, so that the model can more accurately reflect the freezing damage in the analysis of the embryo.

**Table 4:** The confusion matrix, sensitivity, Accuracy RND, and ‘Accuracy-Accuracy RND’ results of the optimal wavelength selection model on each side.

Seed Side		Training Set			Testing Set		
		Category 1	Category 2	Category 3	Category 1	Category 2	Category 3
Endosperm side	Category 1	533	0	0	264	1	2
	Category 2	3	392	32	2	191	20
	Category 3	7	49	264	4	27	129
	Sensitivity,%	100	91.8	82.5	98.9	89.7	80.6
	Accuracy,%	92.9			91.2		
	Accuracy RND,%	34.9			34.9		
	Accuracy-Accuracy RND,%	58.0			56.3		
Embryo side	Category 1	533	0	0	267	0	0
	Category 2	0	413	14	0	200	13
	Category 3	2	16	302	1	19	140
	Sensitivity,%	100	96.7	94.4	100	93.9	87.5
	Accuracy,%	97.5			94.9		
	Accuracy RND,%	34.8			34.8		
	Accuracy-Accuracy RND,%	62.7			60.1		

### 2.6. Discussion

From the perspective of model performance, although the full waveband covered the complete spectral information of the sample, the performance of the model was significantly improved by combining the feature wavelength extracted by the SPA and the 2DCOS method, and even exceeded the performance of the model based on the full waveband in some cases. Full-spectrum data contain more redundant information and noise, which will interfere with the model’s learning of key features and affect the model’s generalization ability. As a forward variable selection method, SPA can select uncorrelated but information-rich variable subsets, whereas 2DCOS can detect low-response wavelengths, and the combination of the two can accurately select wavelengths closely related to the characteristics of corn seed freezing damage and build a more efficient and accurate model. Specifically, in the analysis of the endosperm and embryo parts, the feature wavelengths extracted by the SPA+2DCOS algorithm greatly reduces the amount of data, reduces the demand for computing resources, and makes data processing and model training more efficient. Only 19 and 15 selected feature wavelengths are needed to achieve good recognition results.

In terms of modeling methods, the accuracy of the LDA and SVM models used in this study is superior to that of the KNN model in classification performance. With the development of data processing methods, convolutional neural networks (CNNs) show some potential in data processing. Their convolutional layer, pooling layer, and fully connected layer structure can automatically learn complex features in the data and capture the correlation and local features between different wavebands of hyperspectral data without pre-setting the feature wavelength [32,33]. But they need a great deal of training data and high-performance hardware support in practical applications. In this study, these models combined with the feature wavelengths extracted by SPA+2DCOS were able to achieve better classification results, meeting the basic requirements for model performance. Moreover, this study focuses on the fusion algorithm of feature wavebands and expects to obtain more results by fewer data, while CNN directly processes the full wavelength, which does not meet the research needs, and it is difficult to determine the key spectral features. Although indicators such as the confusion matrix and sensitivity were used to evaluate the model performance in this study, it was also possible to visually compare which model has better results. However, in the field of statistics, if there are significance tests to compare the performance between models and preprocessing methods, this will lead to a better evaluation of the results.

Compared with previous studies, this study discussed the effect of freezing damage on the endosperm side of corn seeds and compared the identification performance of the endosperm side and the embryo side. At the same time, through the innovative feature wavelength extraction and fusion algorithm and the reasonable selection of modeling methods, excellent performance on freezing damage detection could still be achieved.

Although this study can fill the gap in different side detection of corn seed freezing damage to some degree, there are also some limitations. For example, the number of seed varieties used in this study was relatively small and cannot represent all corn samples. Although three varieties (Haoyu 21, Haihe 78, and Jindan 10) were introduced to identify freeze-damaged corn seeds [20], this is not completely representative of all corn samples, due to differences in the samples; the varieties of frost tolerance were also different. In future studies, different varieties of corn with cold tolerance may be selected for analysis, related research from physicochemistry to nondestructive testing can be carried out, and the data set of samples can be expanded at the same time, which will help to establish a more general frost damage detection model and perhaps improve the prediction ability and scalability of the model, so as to obtain more excellent and universal results. Meanwhile, a multi-spectral detection system for the rapid nondestructive identification of frozen corn seeds is needed.

When this system is developed, it will bring huge benefits to farmers and the industry. The farmers can eliminate these damaged seeds, avoid the cost of inefficient planting in the field, and greatly reduce the waste caused by using damaged seeds. Compared with other detection methods, the traditional method of seed frost damage detection has a long detection period and low efficiency, and cannot meet the needs of large-scale seed detection. The proposed detection system can detect a large number of seeds in a short period of time and does not cause any damage to the seeds, ensuring the integrity of the seeds and the subsequent sowing value. This advantage provides the system with a broad application prospect in the field of corn seed quality inspection.

## 3. Materials and Methods

### 3.1. Sample Preparation

The corn variety (‘Deyu 997’) was purchased from local farmers in Changchun, Jilin Province, China, and its initial water content was about 25%. The preparation of the freeze-damaged corn samples was consistent with the process of our previous study [32]. That is, the seeds were placed under different environmental conditions, and then the samples were placed in the natural environment. After the water content of the seeds was reduced to 13%, the hyperspectral image data of the seeds were collected. According to our previous study [6], the germination conditions and the biological indicators including the activities of related enzymes (SOD, POD, CAT, and AMS) were different from each other in different frozen corn seeds. In this study, the seeds were divided into 3 categories (800 for normal samples—unfrozen; 640 for slightly freeze-damaged samples—frozen at −5 °C, 10 h and −10 °C, 10 h; 480 for severely freeze-damaged samples—frozen at −15 °C, 10 h and −20 °C, 10 h). The KS algorithm was used to divide the data set according to a ratio of training set to testing set of 2:1. Finally, the sample numbers of each category of the training set and testing set were 533, 427, and 320, and 267, 213, and 160, respectively.

### 3.2. Hyperspectral Image Acquisition and Correction

The hyperspectral data were collected using a VIS/NIR hyperspectral imaging system (Dualix Spectral Imaging, Inc., Wuxi, Jiangsu, China). The system consists of a CCD camera (C8484-05G, Hamamatsu Photonics, Hamamatsu City, Japan), an imaging spectrometer (Impressor V10E-QE, Spectral Imaging Ltd., Oulu, Finland), a lens (V23-f/ 2.4030603, Specim Ltd., Oulu, Finland), a line light source (P/N9130, Illumination Technologies, Inc., New York, NY, USA), a light source controller (2900ER, Illumination Technologies, Inc., USA), a sample station (GZ02DS20, Guangzheng Instruments Co., Ltd., Beijing, China), a mobile platform and its controller (PSA200-11-X, Zolix Instruments Co., Ltd., Beijing, China), a hyperspectral data acquisition software (SpectralCube V2.75b), and a mobile platform control software.

The steps of hyperspectral image data acquisition were as follows:

(1) Parameter determination: SpectralCube V2.75b software was used to collect hyperspectral image data, which were used to achieve excellent imaging status by adjusting the intensity of the light source, the object distance, etc. The final parameters were set as follows: the object distance was 28.5 cm, and the moving speed of the mobile station was 2.6 mm/s. A 0.5 mm extension tube was added to obtain a clearer hyperspectral image of corn seeds.

(2) Black and white correction of the system: After the parameters were determined, black and white correction was carried out in order to overcome the uneven intensity of the light source and the influence of the dark current of the sensor. Firstly, the lens was covered with the lens cap and the light source was turned off to collect the dark-field hyperspectral image data *I_B_*. Then, the light source was turned on, the Teflon white board was placed on the sample table, and the hyperspectral image data *I_W_* were collected.

(3) Hyperspectral data collection. After the black and white correction was conducted, the samples were placed on the sample stage with the endosperm and embryo side, and the hyperspectral data *I_o_* of different frozen corn seeds were collected in the range of 400–1000 nm, with a total of 477 wavelengths. The hyperspectral image data I were corrected according to Equation (1):

(1) $$I=\frac{I_{o}-I_{B}}{I_{W}-I_{B}}\;\;\;\;\;$$

where I are the calibrated data, *I_o_* are the original collected hyperspectral data, *I_B_* are the dark-field hyperspectral data, *I_W_* are the Teflon white-board hyperspectral data.

### 3.3. Data Processing Methods

#### 3.3.1. Imaging Segmentation and Spectrum Extraction

**Figure 5:**
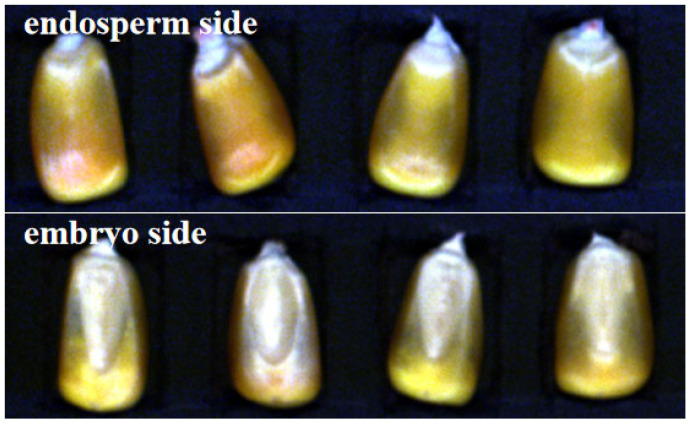
The endosperm-side and embryo-side of corn seeds.

ENVI 4.6.1 (ITT Visual Information Solutions, Boulder, CO, USA) was used for analyzing and processing the hyperspectral image data. The optimal segmentation wavelengths of the region of interest (ROI) of each endosperm and embryo region were segmented from the seed and black background. Figure 5 shows the endosperm and embryo side of corn seeds.

According to our previous study [17,20], image enhancement and the Otsu threshold segmentation procedure were conducted on the gray image at 700 nm and 500 nm, and the endosperm and embryo hyperspectral images were obtained, respectively. Due to the large number of pixels on the endosperm and embryo regions, the pixel spectra in each endosperm and embryo region were averaged, and the average spectrum of each seed was used for spectral analysis. Due to the low response and high noise of the spectrometer, some spectral wavelength data before and after should be eliminated, and the spectra in the 36–455 waveband range (from 450 to 979 nm) were finally selected for analysis.

#### 3.3.2. Spectrum Preprocessing Methods

In this study, the standard normal variation (SNV) and 5 points and 3 times smoothing (5-3 smoothing) preprocessing methods were applied [20].

SNV is mainly used to eliminate interference by solid particle size, surface scattering effects, and optical path variations on spectral data. In order to reduce the influence of interference factors and improve the smoothness of the spectral curve, the 5-3 smoothing method is used to smooth sampling points by 5 points and 3 times polynomial least square fitting. The smoothing time was 2000. This method has a good filtering effect [21,22].

#### 3.3.3. Feature Wavelength Extraction Methods

The successive projections algorithm (SPA), two-dimensional correlation analysis (2DCOS), and the combined wavelength of these two methods were applied. SPA is a forward variable selection method that can select an unrelated and information-rich subset of variables among many variables (wavelengths). It is an algorithm that selects the feature wavelength by analyzing the vector projection, that is, by selecting the wavelength with the largest projection vector. It aims to find the combination of variables with the least redundant information in the variable matrix in order to reduce the collinearity between variables [23,24,25]. The wavelength selection range of SPA is set to the range of 1 to 20. 2DCOS is a mathematical tool used to analyze spectral data sets affected by external interference to identify critical peaks. It can detect low-response wavelengths, which may reveal the key feature wavelengths in the field of seed frost damage recognition and classification. The researchers use this method to automatically generate two-dimensional correlation synchronization spectra and extract characteristic wavelengths from them [29]. As for the better results obtained by SPA and 2DCOS, it may be that these methods can select the wavelength closely related to the characteristics of freezing damage of corn seeds, so that the final selected wavelengths can better represent the real characteristics of freezing damage.

The freezing damage conditions were regarded as an external disturbance, and the feature wavelengths were analyzed by using two-dimensional correlation near-infrared spectroscopy. By generating a two-dimensional correlation synchronization spectrum based on external disturbance, the automatic peak on the diagonal was extracted, and the wavelengths were obtained by analyzing the peak value on the curve formed by the automatic peak.

#### 3.3.4. Classification Models

Three models (K-Nearest Neighbors (KNN), Linear Discriminant Analysis (LDA), and Support Vector Machine (SVM)) were established for the classification of freeze-damaged corn seeds.

KNN is a common classification algorithm in machine learning. Firstly, the distance between the testing sample and each training sample is calculated, and the distance is sorted by increasing order. Then, the k points with the smallest distance are selected to determine the frequency of the corresponding category. Finally, the category with the highest frequency among the first k points is returned as the final classification category of the testing sample [34]. LDA projects the data in a low dimension. It assumes that the data conform to Gaussian distribution and thus LDA projection is conducted. Then, the mean and variance of the projected data are obtained by maximum likelihood estimation, and the probability density function of Gaussian distribution is obtained. Next, the new sample is projected and the probability belonging to each Gaussian distribution is calculated with its characteristics; the highest probability is the sample prediction category; the number of components for LDA is set to 2 [35]. SVM is an important classification method, which has advantages in solving nonlinear and high-dimensional pattern recognition problems in small sample sets. It is based on the VC dimension theory and the structural risk minimization principle of statistical learning theory, and attempts to improve the generalization ability of a learning machine by building models from limited training samples and obtaining small errors for independent test sets; radial basis function (RBF) was applied in the SVM model [36].

After the models are established, the modeling result is represented by Accuracy, which refers to the ratio of the number of correctly classified samples to the total number of samples [37]. The calculation formula is given by Equation (2):

(2) $$Accuracy=\frac{TP+TN}{TP+TN+FP+FN}\times 100\%$$

When the optimal wavelength selection model of each side is obtained, the confusion matrix and sensitivity are applied to compare model performance. Sensitivity refers to the correct classification ability of the model for normal samples. The calculation formula is given by Equation (3):

(3) $$Sensitivity=\frac{\mathrm{TP}\;}{TP+FN}\times 100\%\;$$

‘Accuracy RND’ is an important index to evaluate model quality [38]. The calculation formula is given by Equation (4):

(4) $$AccuracyRND=\frac{\sum_{i=1}^{3}\left(TP+FP)\times (TP+TN\right)}{N\times N}\times 100\;\%\;\;\;\;\;\;$$

In addition, ‘Accuracy—AccuracyRND’ is also a useful index to evaluate the model quality [38]. Its calculation formula is Equations (2)–(4).

True positive (TP): a normal sample is judged as a normal sample; false positive (FP): an abnormal sample is judged as a normal sample; true negative (TN): an abnormal sample is judged as an abnormal sample; false negative (FN): a normal sample is judged as an abnormal sample. In our study, the N values of the training set and testing set were 1280 and 640, respectively.

## 4. Conclusions

The aim of this study was to explore the effect of freezing damage detection on the endosperm and embryo sides of single corn seeds based on hyperspectral imaging and a fusion algorithm. This study compared and analyzed the modeling accuracy results with preprocessing methods and feature wavelength extraction methods (including wavelength fusion) under three models. Moreover, the confusion matrix and sensitivity results of the optimal wavelength selection methods on each side were discussed.

In terms of endosperm-side freezing damage identification, the highest accuracy based on the full-waveband data appeared in the combined SNV+SVM model, and the highest accuracy of the training set and testing set reached 92.9% and 90.0%, respectively. The SNV+SPA-2DCOS+SVM model based on feature wavelengths also performed well, with the highest accuracy of the training set and testing set reaching 92.9% and 91.2%, respectively. In terms of embryo-side freezing damage identification, the 5-3 smoothing+LDA model based on full-waveband data (with a training set and testing set accuracy of 97.7% and 95.9%), as well as the none+SPA-2DCOS+LDA model based on feature wavelengths (with a training set and testing set accuracy of 97.7% and 95.9%), achieved the highest accuracy results. When the fusion algorithm of SPA and 2D-COS was used, the model effect of the endosperm side could be better than that of the full-waveband analysis with only 19 feature wavelengths, while the recognition effect of the embryo side could be achieved with only 15 feature wavelengths. Compared with the results on both sides, the results of the embryo side of the corn seed were better than those of the endosperm side, which indicated that embryo-side data could reflect the freezing damage of seeds more accurately to a certain extent.

These results provide a theoretical basis for constructing a multi-spectral detection system for the rapid and nondestructive identification of frozen corn seeds, effectively reducing the computational burden of full-waveband analysis, and provide support for the online detection of frozen seeds.

## Figures and Tables

**Table 1:** The wavelengths obtained by each feature extraction algorithm under different preprocessing methods.

None	Endosperm Wavelength/nm (Number)	Embryo Wavelength/nm (Number)
Full-band	450–979 (420)	450–979 (420)
SPA	839, 903, 949, 979 (4)	450, 454, 467, 490, 504, 538, 636, 686, 800, 859, 891, 979 (12)
2DCOS	743, 848, 974 (3)	744, 848, 974 (3)
SPA+2DCOS	743, 839, 848, 903, 949, 974, 979 (7)	450, 454, 467, 490, 504, 538, 636, 686, 744, 800, 848, 859, 891, 974, 979 (15)
SNV	Endosperm Wavelength/nm (Number)	Embryo Wavelength/nm (Number)
Full-band	450–979 (420)	450–979 (420)
SPA	509, 523, 586, 705, 738, 770, 809, 820, 873, 879, 886, 904, 921 (13)	509, 520, 554, 640, 703, 743, 800, 876, 886, 911, 922, 938 (12)
2DCOS	467, 589, 676, 745, 848, 899 (6)	580, 676, 745, 846, 899 (5)
SPA+2DCOS	467, 509, 523, 586, 589, 676, 705, 738, 745, 770, 809, 820, 848, 873, 879, 886, 899, 904, 921 (19)	509, 520, 554, 580, 640, 676, 703, 743, 745, 800, 846, 876, 886, 899, 911, 922, 938 (17)
5-3 smoothing	Endosperm Wavelength/nm (Number)	Embryo Wavelength/nm (Number)
Full-band	450–979 (420)	450–979 (420)
SPA	550, 576, 638, 696, 736, 832, 864, 949, 961 (9)	450, 470, 487, 511, 532, 562, 700, 740, 825, 854, 876, 949, 962, 973 (14)
2DCOS	738, 850 (2)	742, 850 (2)
SPA+2DCOS	550, 576, 638, 696, 736, 738, 832, 850, 864, 949, 961 (11)	450, 470, 487, 511, 532, 562, 700, 740, 742, 825, 850, 854, 876, 949, 962, 973 (16)

**Table 3:** The accuracy results of each model under different preprocessing methods on the embryo side.

Preprocessing	Extraction Methods	KNN		LDA		SVM	
		Training	Testing	Training	Testing	Training	Testing
None	Full-band	94.2%	89.1%	96.6%	93.3%	97.3%	94.1%
	SPA	94.5%	89.9%	97.3%	94.4%	96.6%	93.1%
	2DCOS	92.0%	88.2%	93.9%	89.7%	93.6%	89.6%
	SPA+2DCOS	94.7%	90.3%	97.5%	94.9%	96.7%	93.2%
SNV	Full-band	93.9%	91.2%	96.9%	93.0%	98.1%	95.4%
	SPA	93.1%	89.8%	95.0%	92.3%	96.7%	93.1%
	2DCOS	90.5%	85.9%	93.0%	87.2%	93.1%	87.2%
	SPA+2DCOS	94.1%	89.5%	95.0%	92.2%	96.7%	94.1%
5-3 smoothing	Full-band	94.1%	88.7%	97.7%	95.9%	97.3%	94.0%
	SPA	94.4%	89.6%	97.7%	94.8%	97.2%	93.8%
	2DCOS	66.8%	70.3%	80.2%	75.8%	80.3%	75.8%
	SPA+2DCOS	95.0%	89.8%	97.7%	94.8%	97.2%	93.8%

## Data Availability

The original contributions presented in this study are included in the article. Further inquiries can be directed to the corresponding author.
